# Supplementary material for: Multiple Fingerprints and Spectrum-Effect Relationship of Polysaccharides from Saposhnikoviae Radix
Source: Molecules. 2022 Aug 18;27(16):5278. doi: 10.3390/molecules27165278 (PMC9415499; doi:10.3390/molecules27165278)
Supplement: Supplementary file 1 [file molecules-27-05278-s001.zip › molecules-1826140-supplementary.pdf]

Table S1. 10 batches of Saposhnikoviae Radix.

Batche	Origin	Specifications	Batch number	Harvest time	Sourcing company
S1	Heilongjiang	Wild varieties	90351102	2019-11-21	Sanhe
S2	Heilongjiang	Planting varieties	90350901	2019-09-27	Sanhe
S3	Inner Mongolia	Wild varieties	191209	2019-12-09	Crane extension of age
S4	HeBei	Planting varieties	190917001	2019-09-17	Tongrentang, Beijing
S5	Inner Mongolia	Wild varieties	200101	2020-01-01	Quanzhou heron yan
S6	Inner Mongolia	Wild varieties	200509	2020-05-09	Quanzhou heron yan
S7	Ji Lin	Wild varieties	2001046	2020-01-26	Build yanjing
S8	Ji Lin	Wild varieties	2005009	2020-05-19	Build yanjing
S9	Ji Lin	Wild varieties	19070801	2019-07-08	Tongrentang, Beijing
S10	Inner Mongolia	Wild varieties	191223004	2019-12-23	De Shoutang, Beijing

Table S2.1. similarity evaluation of PMP-HPLC chromatogram of SPs.

batch	Similarity coefficient	Similarity(cos θ)
S1	0.953	0.983
S2	0.970	0.989
S3	0.962	0.986
S4	0.998	0.999
S5	0.990	0.996
S6	0.983	0.991
S7	0.999	0.999
S8	0.992	0.997
S9	0.980	0.992
S10	0.957	0.985

Table S2.2. PMP-HPLC chromatographic component matrix of 10 batches of SPs.

Peak number	Principal component 1	Principal component 2
1	0.898	-0.154
2	0.604	0.674
3	0.265	0.640
4	0.897	-0.345
5	0.815	0.421
6	0.705	-0.669

Table S3.1. Molecular weight distribution of HPSEC chromatogram of SPs.

batch	Peak number	Mn (Da)	Mw (Da)	Mz (Da)	Peak area (%)	PDI (Mw/Mn)
	1	9.16×10^6	9.39×10^6	9.58×10^6	87.37	1.025194
1	2	2.55×10^6	3.08×10^6	3.47×10^6	3.17	1.20993
	3	3.17×10^3	1.08×10^4	2.92×10^4	9.46	3.41193
	1	9.45×10^6	9.65×10^6	9.83×10^6	90.74	1.022104
2	2	3.00×10^6	3.39×10^6	3.69×10^6	3.05	1.126895
	3	3.38×10^3	1.14×10^4	2.87×10^4	6.21	3.358145
	1	8.67×10^6	8.91×10^6	9.13×10^6	90.5	1.027879
3	2	2.63×10^6	3.02×10^6	3.30×10^6	3.43	1.146059
	3	3.57×10^3	8.89×10^3	1.93×10^4	6.07	2.48834
	1	9.07×10^6	9.28×10^6	9.46×10^6	87.83	1.023166
4	2	2.63×10^6	3.03×10^6	3.34×10^6	4.79	1.152343
	3	2.96×10^3	8.10×10^3	1.89×10^4	7.38	2.733397
	1	9.20×10^6	9.40×10^6	9.57×10^6	86.2	1.021402
5	2	2.83×10^6	3.20×10^6	3.49×10^6	5.38	1.130644
	3	3.91×10^3	1.15×10^4	3.32×10^4	8.43	2.932875
	1	9.56×10^6	9.74×10^6	9.89×10^6	87.66	1.018703
6	2	3.14×10^6	3.50×10^6	3.78×10^6	4.86	1.112845
	3	3.76×10^3	1.03×10^4	2.48×10^4	7.47	2.758124
	1	9.07×10^6	9.29×10^6	9.47×10^6	80.39	1.023905
7	2	2.50×10^6	3.07×10^6	3.46×10^6	5.5	1.226278
	3	2.10×10^3	1.50×10^4	5.91×10^4	14.1	7.144441
	1	9.29×10^6	9.50×10^6	9.68×10^6	81.44	1.022441
8	2	2.56×10^6	3.14×10^6	3.53×10^6	4.4	1.230394
	3	1.69×10^3	1.02×10^4	3.80×10^4	14.15	6.040636
	1	9.23×10^6	9.44×10^6	9.61×10^6	86.75	1.02239
9	2	2.93×10^6	3.22×10^6	3.46×10^6	4.76	1.097743
	3	4.00×10^3	1.19×10^4	3.51×10^4	8.49	2.971091

	1	9228053	9477680	9686274	82.66	1.027051
10	2	2635254	3254353	3685284	5.26	1.234929
	3	3178	14281	41640	12.08	4.493101

Table 3.2. similarity evaluation of HPSEC chromatogram of SPs.

batch	Similarity coefficient	Similarity(cos θ)
S1	0.999	0.999
S2	1	1
S3	0.999	0.999
S4	1	1
S5	1	1
S6	0.999	0.999
S7	1	1
S8	0.998	0.999
S9	1	0.999
S10	1	1

Table S3.3. HPSEC chromatographic component matrix of 10 batches of SPs.

Peak number	Principal component 1	Principal component 2
1	0.785	0.119
2	−0.443	0.880
3	0.730	0.407

Table S4.1. similarity evaluation of FT-IR chromatogram of SPs.

batch	Similarity coefficient	Similarity(cos θ)
S1	0.959	0.998
S2	0.963	0.992
S3	0.976	0.993
S4	0.992	0.999
S5	0.993	0.999
S6	0.991	0.999
S7	0.991	0.991
S8	0.973	0.985
S9	0.994	0.999
S10	0.994	0.989

Table S4.2. FT-IR chromatographic component matrix of 10 batches of SPs.

Wave number（cm^{-1}）	Principal component 1	Principal component 2	Principal component 3	Principal component 4
3389	0.986	-0.138	-0.067	0.069
2935	0.967	-0.238	0.076	-0.014
1743	0.996	-0.0658	0.039	0.01
1621	0.995	-0.083	0.039	0.021
1423	0.998	-0.014	0.046	-0.026
1374	0.999	0.011	0.043	-0.001
1238	0.998	0.05	0.006	-0.007
1079	0.996	-0.01	-0.089	-0.023
1024	0.994	-0.023	-0.104	-0.034
892	0.702	0.711	0.016	0.011
